# A bootstrap approach for assessing the uncertainty of outcome probabilities when using a scoring system

**DOI:** 10.1186/1472-6947-10-45

**Published:** 2010-08-26

**Authors:** Gabriele Cevenini, Paolo Barbini

**Affiliations:** 1 Department of Surgery and Bioengineering, University of Siena, Siena, Italy

## Abstract

**Background:**

Scoring systems are a very attractive family of clinical predictive models, because the patient score can be calculated without using any data processing system. Their weakness lies in the difficulty of associating a reliable prognostic probability with each score. In this study a bootstrap approach for estimating confidence intervals of outcome probabilities is described and applied to design and optimize the performance of a scoring system for morbidity in intensive care units after heart surgery.

**Methods:**

The bias-corrected and accelerated bootstrap method was used to estimate the 95% confidence intervals of outcome probabilities associated with a scoring system. These confidence intervals were calculated for each score and each step of the scoring-system design by means of one thousand bootstrapped samples. 1090 consecutive adult patients who underwent coronary artery bypass graft were assigned at random to two groups of equal size, so as to define random training and testing sets with equal percentage morbidities. A collection of 78 preoperative, intraoperative and postoperative variables were considered as likely morbidity predictors.

**Results:**

Several competing scoring systems were compared on the basis of discrimination, generalization and uncertainty associated with the prognostic probabilities. The results showed that confidence intervals corresponding to different scores often overlapped, making it convenient to unite and thus reduce the score classes. After uniting two adjacent classes, a model with six score groups not only gave a satisfactory trade-off between discrimination and generalization, but also enabled patients to be allocated to classes, most of which were characterized by well separated confidence intervals of prognostic probabilities.

**Conclusions:**

Scoring systems are often designed solely on the basis of discrimination and generalization characteristics, to the detriment of prediction of a trustworthy outcome probability. The present example demonstrates that using a bootstrap method for the estimation of outcome-probability confidence intervals provides useful additional information about score-class statistics, guiding physicians towards the most convenient model for predicting morbidity outcomes in their clinical context.

## Background

Many models to predict the risk of adverse health events have recently been proposed in a wide range of medical fields. Their main goal has been to help physicians in clinical management and/or follow-up of critical patients and also to improve the quality of care, to teach and train inexperienced medical staff and for educational purposes, such as worksite health promotion programs [1].

Two different approaches are employed to define predictive risk rules. The first uses probability models based on logistic regression, Bayesian classification or artificial neural networks, which enable estimation of diagnostic probability, usually also providing reliable individual prognostic information [2-6]. The second utilizes very simple scoring systems, in which the predictor variables are usually selected and scored subjectively by expert consensus or objectively using statistical methods [7,8]. Scoring systems are frequently preferred by clinicians and health operators because they are so simple that individual scores can be assessed immediately, without using any data processing system. For example, they are often employed to help physicians select treatment options and allocate resources in intensive care units, where control of vital functions is the most important goal. Scoring systems are therefore currently used in many clinical applications, despite the fact that they are generally less accurate than probability models and fail to provide precise individual risk estimation. However, since they rely on identifying cut-off values to dichotomize quantitative variables and their trustworthiness depends on the availability a sufficient proportion of adverse outcomes for their design, their correct design is essential for a reliable interpretation of the scores obtained [4,9].

A common problem is the effective exportability of any risk-prediction model to clinical scenarios different from those in which the model was designed; predictive models must be locally validated and tuned to provide risk-adjusted outcomes [3,4,9]. Model customization is often indispensable because standardization of local practices is difficult and patient populations may differ [10-12].

Another important component of model accuracy is agreement between predicted probabilities and observed proportions, known as calibration, which allows a model's prognostic ability to be evaluated [5]. For dichotomous outcomes concerning the presence (or absence) of an adverse health event, true risk probabilities cannot be known intrinsically. Nevertheless, it is sometimes useful to estimate the occurrence of these events using a continuous scale. In particular, an estimated probability of the patient's outcome is usually preferred to a simpler binary decision rule. Model calibration is independent of discrimination, since there are models with good discrimination but poor calibration. A well-calibrated model gives probabilities that can be reliably associated with the true risk of incurring outcomes. While for probability models, calibration can be assessed using the Hosmer-Lemeshov test [13], this test may not be completely appropriate for models with discrete outputs (such as scoring systems).

In this study we describe an approach that can be used for the statistical interpretation of diagnostic and prognostic ability of simple scoring systems. It uses a numerical bootstrap technique to estimate the confidence interval of the risk probability associated with an integer score [14-16]. We use the method to design and optimize the performance of a predictive scoring system for morbidity of heart surgery patients in an intensive care unit (ICU).

## Methods

### Training and testing samples

To describe the method used to assess and optimize the performance of a predictive scoring system, we used the experimental data of a previous study [9], based on a set of 1090 consecutive adult patients who underwent coronary artery bypass grafting and were admitted to the intensive care unit of the Department of Surgery and Bioengineering of Siena University. A collection of 78 preoperative, intraoperative and postoperative variables were considered as likely risk predictors that could be associated with the development of morbidity in the ICU. A detailed description of these variables can be found in the paper by Cevenini et al. [9].

A dichotomous (binary) variable was chosen as ICU outcome (morbidity). Morbidity outcome was defined for patients developing at least one of the following clinical complications: cardiovascular, respiratory, neurological (central nervous system), renal, infectious and hemorrhagic complications. The percentage morbidity in the whole patient set was 20.7%.

Normal and morbid cases were assigned at random to two groups of equal size (545 cases each) so as to define random training and testing sets with equal percentage morbidities. To ensure that this allocation of cases did not introduce systematic sampling errors, training and testing data was compared using the Fisher exact test for dichotomous variables and the z-test or Mann-Whitney test for continuous normally or non-normally distributed variables, respectively [17]. No significant difference was found between training and testing data, setting statistical significance at a p-value less than 0.05.

### Scoring system

The model used to assess cardiac postoperative morbidity was a simple scoring system in which the integer score of morbidity risk was obtained by summing a weighted combination of selected binarized predictor variables [4]. In a previous paper this scoring system was compared to other types of models when applied to prediction of patient outcomes in the ICU [9]. Besides having discrimination similar to various other methods (the Bayes rule, logistic regression, k-nearest neighbour and artificial neural networks), its performance was very similar to the Higgins scoring model, which is a well-known and extensively employed scoring system in post-operative ICUs [18].

The variables were first made dichotomous and then coded with binary values (0 or 1), comparing original values with set cut-off points. Once a cut-off point was selected for a given variable, the resulting *2 × 2 *classification matrix of training data enabled sensitivity and specificity to be computed [17]. Of course, sensitivity and specificity both varied with changes in cut-off point. In the present study, the cut-off point for each variable was chosen as the point of the ROC (receiver operating characteristic) curve closest to the upper left corner (100% sensitivity, 100% specificity).

The discrimination power of each binary-coded variable was then evaluated on the basis of the corresponding 95% confidence interval (CI) of the odds ratio [17,19]. Only binary variables with an odds ratio significantly greater than 1 (p < 0.05) on the training data were considered likely to be chosen as potential morbidity predictors during selection of model features by a forward stepwise algorithm.

At the first step, the forward algorithm of feature selection chose the binary variable with the highest area under the ROC curve (AUC) on the training data. At any subsequent step the variable giving the highest increment to AUC was entered. Various stopping criteria accounting for increments to AUC were defined on the training data from one step to another [20]. Too restrictive a criterion risked stopping the algorithm of variable selection when only a few predictors had been entered in the scoring system, thus reducing the possibility of associating an effective probability of morbidity with each integer score. On the other hand, conclusive selection of the optimal predictor set was also made on the basis of the testing data, optimizing the capacity of the model to maintain good predictive performance on data not used for training. Thus we used a rather soft stopping criterion on the training data, so that the forward procedure stopped when the cumulative increment in AUC obtained in five consecutive steps was less than 1%. The model integer score was simply computed by summing the binary values of the selected variables. At each step, formerly selected variables were also reconsidered for entry in the model. This allowed the model to assign different weights (scores) to each predictor variable by adding its corresponding binary value several times.

The model score for a generic test patient was simply:

(1) $$s=\sum_{i=1}^{d}\;\lambda_{i}\;s_{i}$$

where *d *is the number of predictors in the model, *s*_*i *_the score associated with the *i*^th ^predictor and *λ*_*i *_a coefficient assigned a value of 0 or 1 after comparison of the *i*^th ^predictor with its corresponding cut-off point.

### Confidence interval estimation

In general, a confidence interval for an unknown parameter *θ *is more informative than a point estimate for *θ *alone. The construction of confidence intervals is an area in which the bootstrap has achieved major success, and several techniques are available [15,21].

Bootstrapping is a highly computer-intensive statistical procedure for estimating the sampling distribution of an estimator by sampling with replacement from the original sample. It can be used to produce good approximate confidence intervals when the statistical distribution is unknown or so complex that conventional techniques are not valid and no additional samples are available [15]. The key to the strategy is to create alternative versions of data that "we might have seen". Instead of generating observations from a known theoretical distribution, we generate observations from the distribution of the sample itself - the empirical distribution. After all, available data gives us a lot of information about the relative probabilities of different values, and in certain senses this empirical distribution is actually the least prejudiced estimate possible of the underlying distribution - anything else imposes biases or preconceptions, which are possibly accurate but also potentially misleading. Each simulation results in a new sample, typically of the same size as the original, by randomly selecting (with replacement) individuals from the original sample. "With replacement" means that at each step in the selection process, every individual from the original sample is again eligible to be selected, whether or not he/she has already been selected. Thus, in each bootstrap sample, some of the original individuals may not be represented and others may be represented more than once.

With respect to other numerical methods, bootstrapping methods show a lower bias or variance and exploit computer processing power to estimate even complex statistical parameters in a simpler way than analytical methods [15,16,21].

A natural question when employing the bootstrap approach to estimate confidence intervals concerns the number of bootstrap samples needed to achieve accurate intervals. Based mainly on empirical evidence, several researchers [14] have reported that one thousand bootstrap samples are enough to accurately estimate bootstrap confidence intervals.

The percentile interval is the simplest bootstrap method for calculating a confidence interval. To calculate a 95% confidence interval it is enough to select the bootstrap estimates which lie on the 2.5^th ^percentile and 97.5^th ^percentile. The percentile intervals are very simple to use but comparison with exact statistical methods have shown them to have unsatisfactory coverage in some cases. Part of the problem with percentile confidence intervals is that the bootstrap estimates are biased with respect to the original estimate and the standard error varies with the value of the estimate. Consequently the percentile bootstrap has been extended in many different ways to increase confidence accuracy [22].

The bias-corrected and accelerated (BCa) bootstrap method is considered a substantial improvement over the standard percentile method [21]. It employs two coefficients, called *bias correction *and *acceleration*, to incorporate information on bias and change in standard error of the estimator into the estimation procedure. An approximate BCa confidence interval is second-order accurate, i.e. its coverage probability differs from its advertised coverage probability by terms which go to zero at a rate of *n*^-1 ^where *n *is the sample size in the case of independent identically distributed observations [14,22]. On the contrary the standard intervals, obtained by taking the parameter estimator and an estimate of its standard deviation, are first-order accurate (i.e. the difference goes to zero at rate *n*^-½^). Evidently, if the sample size is made sufficiently large, the second-order-accurate method will be superior to the first-order one.

The BCa bootstrap intervals are not only asymptotically more accurate than the standard intervals, but they are also more correct [15]. Confidence intervals are said to be correct when they are optimal in the sense of being the shortest possible for the given coverage [23].

In the present study the BCa bootstrap method was used to estimate the confidence intervals of the prognostic probabilities associated with estimated integer scores [14,15]. The 95% confidence intervals of patient morbidity percentages were calculated for each score and each step of model design, using one thousand bootstrapped samples generated from the training data. All computations were made using MATLAB code.

## Results

**Figure 1:**
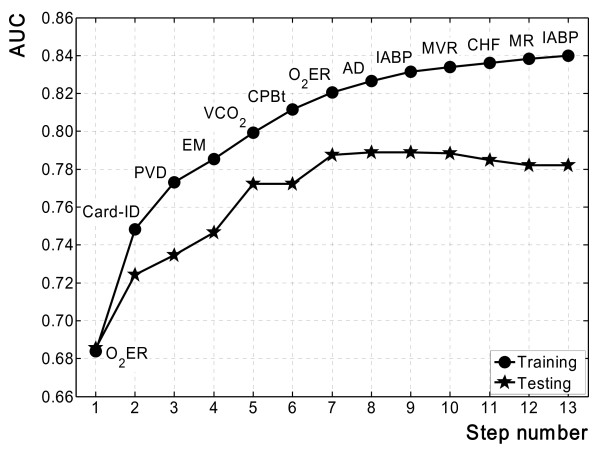
**AUC calculated with training (circles) and testing data (stars) for each step of the forward stepwise algorithm of feature selection performed on the training set**. The predictor entered is also reported step by step.

The circles in Figure 1 show the AUC values obtained using the above procedure for the forward selection of model features on the training data. The stopping criterion arrested the stepwise algorithm at the thirteenth step, after eleven predictor variables were selected: two variables (O_2_ER and IABP) entered twice.

Table 1 summarizes the whole set of variables selected on the basis of training data only. Three variables referred to preoperative information about congestive heart failure (CHF), peripheral vascular disease (PVD) and emergency (EM). All these variables were intrinsically dichotomous. In particular, emergency cases were defined as unstable angina, unstable hemodynamics, or ischemic valve dysfunction that could not be controlled medically [18]. Four selected variables were related to surgery features: mitral valve replaced with artificial valve (MVR) or repaired surgically (MR), intra-aortic balloon pump (IABP) and cardio-pulmonary bypass time (CPBt). While the first three surgical variables were intrinsically dichotomous, the last one was made dichotomous setting a cut-off point equal to 2 hours, i.e. CPBt ≥2 hours was considered a morbidity predictor. Finally, the remaining four variables were postoperative data collected in the first three hours after admission to the ICU. Cardiac inotropic drugs (Card-ID) and anti-arrhythmic drugs (AD) were intrinsically dichotomous, whereas oxygen extraction ratio (O_2_ER) and carbon dioxide production (VCO_2_) were made dichotomous (O_2_ER ≥40% and VCO_2 _< 180 ml/min were taken as morbidity predictors).

**Table 1:** Dichotomous variables entered in the score model

Acronym	Description	Training N (%)	Testing N (%)
O_2_ER	O_2 _extraction ratio ≥40%	116 (21.3%)	129 (23.7%)

Card-ID	Cardiac inotropic drugs	74 (13.6%)	69 (12.7%)

PVD	Peripheral vascular disease	116 (21.3%)	104 (19.1%)

EM	Emergency	45 (8.3%)	48 (8.8%)

VCO_2_	CO_2 _production <180 ml/min	256 (47.0%)	261 (47.9%)

CPBt	Cardio-pulmonary bypass time ≥2 hours	223 (40.9%)	203 (37.2%)

AD	Anti-arrhythmic drugs	29 (5.3%)	27 (5.0%)

IABP	Intra aortic balloon pump	11 (2.0%)	10 (1.8%)

MVR	Mitral valve replaced with artificial valve	8 (1.5%)	12 (2.2%)

CHF	Congestive heart failure	25 (4.6%)	30 (5.5%)

MR	Mitral valve repaired surgically	12 (2.2%)	8 (1.5%)

The stars in Figure 1 indicate the AUC values calculated, step by step, from testing data using the model developed on the training data. Although we see that AUC obtained on training data continued to go up until the last step, no appreciable increase in AUC in the testing data was observed after step 7, after which model generalization worsened. Actually, a reasonable compromise between discrimination and generalization seemed to be reached at step 7.

If the aim of the scoring system is not only assessment of a risk score but also a reliable prediction of the probability of morbidity outcome, the 95% confidence interval of the prognostic probability associated with each integer score has to be calculated extending the analysis to a wider range of steps.

**Figure 2:**
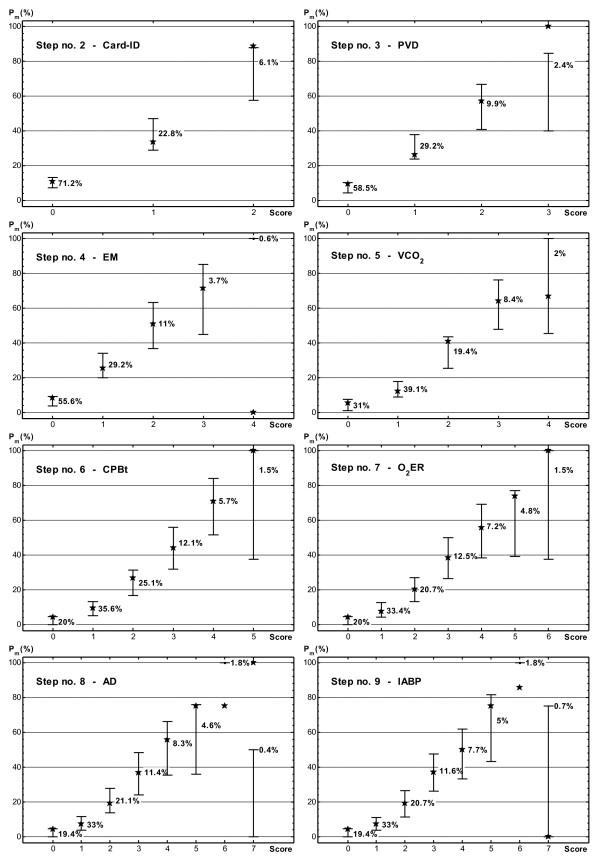
**95% confidence intervals of morbidity probability estimated with the bias-corrected and accelerated bootstrap method from training data for competing scoring systems**. Stars indicate the probability of morbidity calculated on the testing data. The percentage occurrence of each score and the predictor entered at a given step are also reported.

Figure 2 shows the statistical properties of model scores identified by the BCa bootstrap method, from step 2 to step 9. For each step, 95% CI from training data was plotted in a whisker diagram where the corresponding probability of morbidity on testing data (stars) and the percentage occurrence of each score were also reported.

Step 2 shows that the estimated probability of morbidity significantly increases with the score. The scoring system gave three integer scores (0, 1 and 2) when a satisfactory number of patients was observed, the minimum being 33 (6.1%) with the highest score. The risk probabilities calculated on testing data always fell within the corresponding confidence interval of the training data. However, the large separation between two consecutive CIs suggests that other variables (and therefore other score values) could be considered in the model to obtain a finer class division. Moreover, Figure 1 indicates that the discrimination capacity of the scoring system corresponding to step 2 was not good enough.

Of course, inclusion of an additional variable at step 3 led to distribution of patients in four score classes, but the few cases (13 or 2.4%) with the highest score affected CI size, so that a wide overlap was observed between scores of 2 and 3. Besides, the risk probability computed on the testing patient with a score of 3 fell outside the CI obtained from the training data. This evidence suggested combining patients with scores of 2 or 3 in a single class. A larger number of cases would narrow the confidence interval, separating it from the others. Indeed, reduction to three classes (score = 0, score = 1 and scores ≥2) reproduced a scenario not too dissimilar with respect to step 2, although it had the positive effect of balancing the distribution of cases within each class.

Analysis of the results of step 4 led to a conclusion similar to that for step 3. In fact the confidence intervals corresponding to scores of 2 and 3 again overlapped, while only three training cases reached the maximum score.

**Figure 3:**
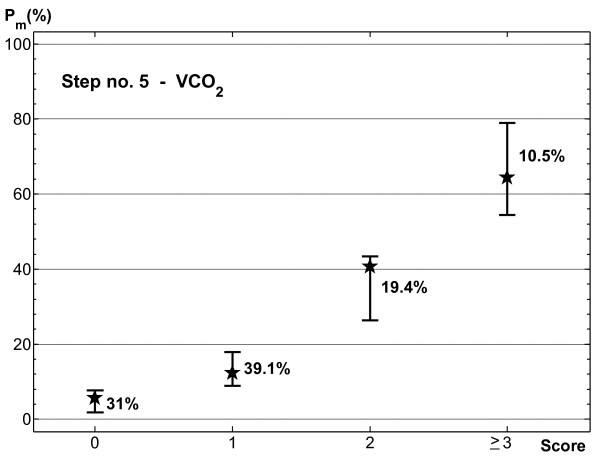
**95% confidence intervals of morbidity probability estimated with the bias-corrected and accelerated bootstrap method for the scoring system with four score classes**. Stars indicate the percentage of morbid patients observed for each score class in the testing data.

The results obtained from step 5 showed interesting enhancement of model prognostic ability. Of course patients having a score of 3 or 4 could be profitably pooled in a single class to eliminate the CI overlap and enlarge class size. This class pooling did not influence the discrimination capacity of the scoring system. No appreciable decrease was observed with respect to the unmodified model when calculating AUC of training and testing data (0.798 and 0.772, respectively). The modified scoring system was characterized by four score classes (0, 1, 2, ≥ 3), thus enabling finer class division than the previous-step models and ensuring good separation of the estimated 95% confidence intervals (see Figure 3). Comparison of the results of the simpler step-2 model with those shown in Figure 3 indicated that the scoring system with four classes effectively split the lowest risk class of the simpler model into two distinct classes associated with very low (less than 10%) and low (between 10% and 20%) probability of morbidity. The change was not so evident for the moderate and high risk classes.

**Figure 4:**
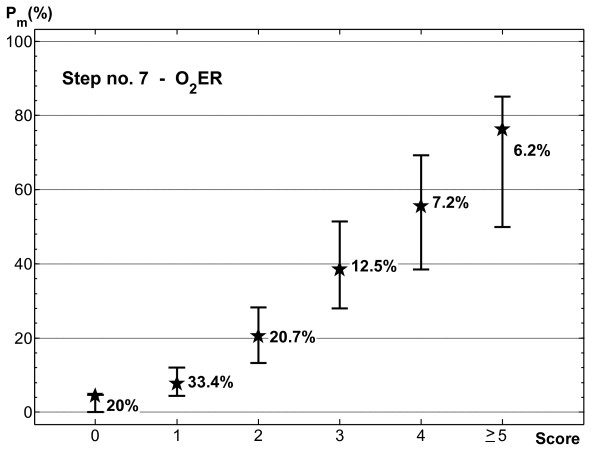
**95% confidence intervals of morbidity probability estimated with the bias-corrected and accelerated bootstrap method for the scoring system with six score classes**. Stars indicate the percentage of morbid patients observed for each score class in the testing data.

Because a probability estimate of a patient's outcome is very useful in the ICU if patients at risk for high morbidity are correctly recognized, it could be worthwhile increasing the number of classes, at the risk of some CI overlap. This can be done by continuing to analyze further steps. Of course the number of cases in each class significantly decreases, especially for higher scores. Figure 2 shows that cases with scores greater than 6 were too few in the sample, so we focused on the results obtained at step 7, which seemed to be a reasonable compromise between discrimination and generalization (Figure 1). Unfortunately, the confidence intervals of the highest scores overlapped considerably, so that patients with scores of 5 or 6 were united in a single risk class. The corresponding scoring system had six score classes: 0, 1, 2, 3, 4 and ≥5. It maintained the same discrimination as the original model with seven classes. For both models AUC was 0.820 when calculated on training data, and decreased negligibly on testing data (from 0.788 to 0.780). Figure 4 shows the 95% confidence intervals of the prognostic probabilities of the six-class model. Patients with scores of 0, 1, 2, 3 and ≥5 had clearly separated confidence intervals for morbidity probabilities. In particular, patients with scores ≥5 had high probability of developing morbidity events in ICU. Alas, patients with score 4 could have a similar probability of morbidity to patients belonging to the two contiguous classes. In summary, this scoring system allowed finer class division than the model of Figure 3 (the number of classes increased from 4 to 6) but the price paid was overlapping of the 95% confidence interval of one patient class with two others. On the basis of the present analysis using the bootstrap technique, the medical team must have sufficient information to choose the best scoring system for the clinical context.

## Discussion

Models for the prediction of patient risk are increasingly used in critical care because they allow diagnostic and prognostic information to be derived precisely and evaluated quantitatively. Scoring systems are a very attractive kind of model in clinical practice, due to their simplicity of application, especially where a rapid and effective decision has to be taken for correct evaluation of patient status.

Despite their simplicity, when carefully designed, the accuracy of scoring systems has proven not sufficiently worse than more complex models, such as logistic regression, Bayesian classification rule or artificial neural networks, to exclude their clinical application [9,18]. A major limit is calibration, i.e. identification of a proper quantitative association between score values and prognostic risk probabilities. Reliable individual prediction of this probability is very important in clinical practice, being a useful tool for medical decision making, patient risk reduction, optimal planning of clinical resources and welfare cost saving.

An idea could be to directly estimate the risk probability by dividing the score of the test patient by the maximum possible score. However, this method may lead to unreliable results and the Hosmer-Lemeshov test (developed for logistic-regression models) may not be appropriate for models with discrete outputs such as scoring systems [13]. Of course, a more straightforward approach is to focus on the statistics of the score classes determined by the model. In the original paper of Higgins and colleagues [18] the risk levels of test patients were categorized on the basis of similar outcomes in the training set. However, an accurate estimate of the uncertainty associated with parameter estimates is important to avoid misleading inference. This uncertainty is usually summarized by a confidence interval, which is claimed to have a specified probability of including the true parameter value. In particular, confidence intervals combine point estimation and hypothesis testing in a single inferential statement of great intuitive appeal. Thus for predictive scoring systems, a crucial point is assessment of the confidence intervals of the estimated risk probabilities.

The bootstrap technique is a resampling method for statistical inference commonly used to estimate confidence intervals [14,15,21]. Although all bootstrap confidence intervals fail to perform well in some situations, this should not overly discourage the use of bootstrap confidence intervals. Some intervals work very well in many situations, and even when they do not work so well, they may still be better than most alternatives. The bootstrap method is more transparent, simpler and more general than conventional approaches. Understanding the rationale behind it does not requires any deep knowledge of mathematics or probability theory. The assumptions on which it depends are less restrictive and more easily checked than the assumptions on which conventional methods depend. The method can be applied to situations where conventional methods may be difficult or impossible to find.

On the basis of the above considerations, we proposed a more informative approach to develop and select competing scoring systems to predict adverse outcomes in medical applications. The model selection not only accounts for discrimination power and generalization of the predictive model, but also for the trustworthiness of the estimated prognostic probability associated with each score class. In particular for each scoring system the 95% confidence intervals of prognostic probabilities are estimated by the BCa bootstrap technique. As an example, the procedure was applied to data collected in heart-surgery patients who underwent coronary artery bypass graft. Since much has happened in the field of heart surgery in recent years, mortality is now low and morbidity has been considered a valid end point and a more attractive target for developing the risk model. The low prevalence of adverse events negatively influences the estimation of confidence intervals for prognostic probabilities, so that end points corresponding to quite rare events, such as death after heart surgery, must be avoided in designing a risk model.

The illustrative example uses a sample of 1090 patients, which was divided into one training set and one testing set of equal size. Cross validation would naturally be a more efficient approach, though more demanding computationally. However, our choice can be considered satisfactory, because sample size was large enough. It also allowed us to easily define training and testing sets with equal percentage morbidities and verify that the random allocation of patients to training and testing did not introduce systematic sampling errors.

The procedure developed enabled us to evaluate and compare several different models in the example considered here. In our opinion, the model with six score classes (Figure 4) has many advantages. First, the scoring system is based on only six variables, two of which give information about preoperative status, one is related to surgery, and the other three are postoperative variables. Despite the low number of predictors, the model shows good discriminating power, also achieving a satisfactory compromise between discrimination and generalization. Finally, it allows patients to be divided into a reasonable number of classes, most characterized by well separated confidence intervals of prognostic probabilities. The only limit of the model is the presence of one score class which has a morbidity-probability confidence interval partially overlapping the two adjacent score classes, so that patients with a score of 4 should be cautiously likened to those with score greater than 4.

Two intrinsically dichotomous preoperative variables (emergency status and peripheral vascular disease) are used in the scoring system. Emergency status is known as a significant preoperative predictor of poor outcome. Emergency patients are more likely to have other risk factors on admission to the ICU, such as low cardiac index, decreased serum albumin, higher alveolar-arterial oxygen gradient, elevated central venous pressure, and tachycardia [18]. Peripheral vascular disease is another important morbidity predictor after coronary artery bypass surgery, especially in predicting severe or mild neurological complications [24].

The postoperative variables are the oxygen extraction ratio (≥40%), carbon dioxide production (< 180 ml/min) and the need for cardiac inotropic drugs after the operation. In particular, the weight of O_2_ER is twice that of the other predictors, indicating a key role for this variable. This important role confirms the results of a previous study, in which increased O_2_ER immediately after heart surgery was indicated as an independent predictor of prolonged ICU stay [25]. O_2_ER reflects a balance between oxygen consumption and oxygen delivery, providing information about compensatory increased extraction in hypovolemia and heart failure.

The only intraoperative variable in the model is cardio-pulmonary bypass time. This variable has been identified as a risk factor in similar studies [3,18]. In particular, the role of CPBt in the determination of hyperlactatemia during cardio-pulmonary bypass has been highlighted by other authors [26,27]. Hyperlactatemia is a well-recognized marker of circulatory failure, and its severity has been associated with mortality in different clinical conditions [28,29]. In particular, high blood lactate levels during cardiopulmonary bypass are associated with tissue hypoperfusion and may contribute to severe postoperative complications. Patients with high blood lactate levels during cardiopulmonary bypass generally need greater and longer hospital care because postoperative morbidity is significantly more frequent [30]. Despite this evidence, the association between hyperlactatemia and postoperative mortality is a much debated question, because different authors have come to different conclusions [30,31]. Finally, data used to develop the scoring system did not account for hyperlactatemia directly but only for CPBt, suggesting that the probability of morbidity may not be properly estimated in off-pump patients.

## Conclusions

When using a scoring system to predict outcomes of critical patients, a proper quantitative association between score values and prognostic risk probabilities is an issue of primary importance. To avoid misleading inference, a trustworthy model should not only ensure good discrimination and generalization but also an accurate estimate of the uncertainty associated with a prognostic probability.

In this paper a bootstrap technique was used to compute the confidence intervals of prognostic probabilities when designing and selecting competing scoring systems. The approach was applied to data of patients who underwent coronary artery bypass grafting to evaluate morbidity risk in the intensive care unit. In the example considered, a model with six score classes showed various advantages with respect to competing scoring systems. Besides having a satisfactory trade-off between discrimination and generalization, the model also allowed patients be divided into a reasonable number of classes, most characterized by well separated confidence intervals of prognostic probabilities. Of course, this does not mean the model is necessarily the best, especially when overlapping of confidence intervals of prognostic probabilities associated with the integer scores must be avoided. However, the example demonstrates that the technique allows useful additional information to be gained about the statistics of the score classes, guiding physicians towards the most convenient model for assessing morbidity in their clinical context.

## Competing interests

The authors declare that they have no competing interests.

## Authors' contributions

All authors participated in the study plan and coordination and contributed to preparation of the manuscript and data processing. All authors read and approved the final manuscript.

## Pre-publication history

The pre-publication history for this paper can be accessed here:

http://www.biomedcentral.com/1472-6947/10/45/prepub
